# HPLC-DAD Phenolic Characterization and Antioxidant Activities of Ripe and Unripe Sweet Orange Peels

**DOI:** 10.3390/antiox4030498

**Published:** 2015-07-09

**Authors:** Olufunmilayo Sade Omoba, Rebeccah Olajumoke Obafaye, Sule Ola Salawu, Aline Augusti Boligon, Margareth Linde Athayde

**Affiliations:** 1 Department of Food Science and Technology, Federal University of Technology, P.M.B. 704, Akure 340284, Nigeria; E-Mail: ajufaye@gmail.com; 2 Department of Biochemistry, Federal University of Technology, P.M.B. 704, Akure 340284, Nigeria; E-Mail: sosalawu@futa.edu.ng; 3 Phytochemical Research Laboratory, Department of Industrial Pharmacy, Federal University of Santa Maria, Build 26, Room 1115, Santa Maria, CEP 97105-900, Brazil; E-Mails: alinebolingon@yahoo.com.br (A.A.B.); marga@ccs.ufsm.br (M.L.A.)

**Keywords:** orange peel, phenolic profile, *in vitro* antioxidant

## Abstract

Phenolic compounds of unripe and ripe sweet orange peels were determined using a high-performance liquid chromatography separation method with diode array detector (HPLC-DAD). The *in vitro* antioxidant properties and the EC_50_ (concentration required to obtain a 50% antioxidant effect) values were also determined. The predominant phenolic compounds were quercitrin, rutin, and quercetin with values of 18.77 ± 0.01 mg/mL, 18.65 ± 0.03 mg/mL, and 10.39 ± 0.01 mg/mL respectively in unripe orange peel and 22.61 ± 0.01 mg/mL, 17.93 ± 0.03 mg/mL, and 14.03 ± 0.02 mg/mL respectively in ripe orange peel. The antioxidant properties revealed 2,2′-azino-bis(3-ethyl benzothiazoline-6-sulfonic acid) diammonium salt (ABTS) scavenging ability of both unripe and ripe orange peels respectively as 14.68 ± 0.01 and 16.89 ± 0.02 mmol TEAC/g, the Ferric Reducing Antioxidant Properties (FRAP) as 70.69 ± 0.01 and 91.38 ± 0.01 mg gallic acid equivalents/100g, total phenol content as 5.27 ± 0.03 and 9.40 ± 0.01 mg gallic acid equivalents/g and total flavonoid content as 3.30 ± 0.30 and 4.20 ± 0.02 mg quercetin equivalent/g. The antioxidant assays showed enhanced potency of extract from ripe orange peel with EC50 values of 2.71 ± 0.03 mg/mL for 2,2-diphenyl-1-picrylhydrazyl (DPPH), 0.67 ± 0.03 mg/mL for hydroxyl radicals (OH*), 0.57 ± 0.02 mg/mL for Fe^2+^ chelation, and 0.63 ± 0.06 mg/mL for malondialdehyde (MDA), and was more potent than unripe orange peel.

## 1. Introduction

In today’s consumer perception of agriculture and food production, aspects like health, safety, and quality have become the key words [1].The uncontrolled production of oxygen-derived free radicals is implicated in the onset of many diseases such as cancer, rheumatoid arthritis, and atherosclerosis, as well as in the degenerative processes associated with aging [2]. Antioxidants are abundant compounds primarily found in fresh fruits and vegetables, and their roles in the prevention of degenerative diseases are emerging. Two basic categories of antioxidants exist, namely, synthetic and natural antioxidants. Synthetic antioxidants are compounds with phenolic structures of various degrees of alkyl substitution, while natural antioxidants can be phenolic compounds (tocopherols, flavonoids, and phenolic acids), nitrogen compounds (alkaloids, chlorophyll derivatives, amino acids, and amines), or carotenoids as well as ascorbic acid [3]. However, the most commonly used synthetic antioxidants such as butylated hydroxyanisole (BHA), butylated hydroxytoluene (BHT), have been alleged of being responsible for liver damage and carcinogenesis [4], thus, the growing interest in natural antioxidants.

*Citrus sinensis*, sweet orange is a fruit with a small evergreen tree (8–15ft), or shrub in the *Rutaceae* (citrus family). It originated from southern China, where it has been cultivated for millennia. Orange is now grown commercially worldwide in tropical, semi-tropical, and some warm temperate regions, and has become the most widely planted fruit tree in the world [5]. Orange is the world’s most popular fruit, and is eaten fresh and used for juice [6]; the fruit is made up of peel, pulp, seeds, and juice. In contrast with other types of fruits, around 34% of the fruit is used for juice production, yielding about 44% of peels as byproducts [7]. The peel and seeds results in a considerable amount of by-products which might be a source of environmental pollution since they are prone to microbial spoilage. Citrus by-products could be of use as functional ingredients in the production of functional foods, since they are good sources of dietary fiber and bioactive compounds [8]. Peel is a good source of phenolic compounds which may potentially be used in food formulations or when extracted can be used as natural antioxidants to prevent oxidation of selected foods [9]. The citrus peel and seeds are very rich in phenolic compounds, such as phenolic acids and flavonoids; the peel is richer in flavonoids than seeds [10]. Flavonoids are a group of natural compounds with variable phenolic structures and are found in plants, they are oxidized by radicals, resulting in a more stable, less-reactive radical. They can be divided into a variety of classes such as flavones (e.g., flavone, apigenin, and luteolin), flavonols (e.g., quercetin, kaempferol, myricetin, and fisetin), flavanones (e.g., flavanone, hesperetin, and naringenin), and others. Several flavonoids such as catechin, apigenin, quercetin, naringenin, rutin, and venoruton are reported for their hapatoprotective activities [11]. Selected flavonoids can directly scavenge superoxides, whereas other flavonoids can scavenge the highly reactive oxygen-derived radical called peroxynitrite. Epicatechin and rutin are also powerful radical scavengers [12]. The scavenging ability of rutin may be due to its inhibitory activity on the enzyme xanthine oxidase. Rutin is also known for its anti-inflammatory and vasoactive properties, as well as for its capability to diminish capillary permeability and to reduce the risk of arteriosclerosis, whereby reducing coronary heart disease, possibly through the diminishing of platelet aggregation [13,14] There are also studies that show a dose-response effect of rutin in inhibiting low-density lipoprotein (LDL) peroxidation [15] and the antioxidant activity of rutin in Fenton reaction [16]. Rutin was suggested as one of the most potent natural inhibitors of advanced glycation endproducts (AGEs) accumulation in human body, suggesting that its anti-glycation activity may mainly be due to its radical scavenging activity [17]. Specific flavonoids are known to chelate iron [18] thereby removing a causal factor for the development of free radicals. Quercetin in particular is known for its iron-chelating and iron-stabilizing properties.

Several authors describe the use of high-performance liquid chromatography with diode array detector (HPLC-DAD) for characterization and quantification of phenolic composition [18,19,20,21,22] The presence of compounds with antioxidant properties in *Citrus sinensis* has been described using HPLC-DAD and UV–Vis spectra [22]. UV detectors are popular among all the detectors because they offer high sensitivity and also because majority of naturally occurring compounds encountered have some UV absorbance, phenolics are frequently identified using UV-VIS detector at wavelengths 190–380 nm [17]. Furthermore, the high sensitivity of UV detection is very important for the compounds of interest that are present in small amounts in a sample [20].

Despite all of the possible uses listed above, citrus peel remains underutilized, and it is treated as waste or as feed to ruminant producing meat in Nigeria. Some earlier reports on the phenol constituents and antioxidant properties of citrus peel are mainly determined on the soluble free phenolic [9,22] which is on the basis of the solvent soluble extraction. Therefore, this study sought to identify and quantify the phenolic compounds in both unripe and ripe orange peels using HPLC-DAD and investigate the *in vitro* antioxidant properties of both peels.

## 2. Experimental Section

### 2.1. Chemicals and Reagents

All chemicals used were of analytical grade for both HPLC analysis and spectrophotometeric analysis. Deoxy-d-ribose, 2,2′-Azino-bis(3-ethylbenzothiazoline-6-sulfonic acid)diammonium salt, 2,2-diphenyl-1-picrylhydrazyl were obtained from Sigma Chemical Company (St. Louis, MO, USA). Methanol, formic acid, caffeic acid, catechin and epicatechin purchased from Merck (Darmstadt, Germany). Naringin (7-[[2-*O*-(6-deoxy-l-mannopyranosyl)-d-glucopyranosyl]oxy]-2,3-dihydro-5-hydroxy-2-(4-hydroxyphenyl)-4H-1-benzopyran-4-one), quercetin (3,3,4,5,7-pentahydroxyflavone), quercitrin (2-(3,4-dihydroxyphenyl)-5,7-dihydro-3-(3,4,5-trihydroxy-6-methyloxan-2-yl)oxychromen-4-one), kaempferol (3,4,5,7- tetrahydroxyflavone), luteolin (3,4,5,7-tetrahydroxyflavone) and rutin (2-(3,4-dihydroxyphenyl)-5,7-dihydroxy-4-oxo-4H-chromen-3-yl6-*O*-(6-deoxy-α-l-mannopyranosyl)-β-d-glucopyranoside) were acquired from Sigma Chemical Company. All other chemicals used were of analytical grade. Distilled water was used for the *in vitro* antioxidant analysis.

### 2.2. Orange Peel Powder Production

Matured sweet oranges (Navel variety) were harvested from a tree in a commercial farm, aged 10 years, the fertilizers used were 2 kg of NPK (Nitrogen, Phosphorus and Potassium, at ratio of 15:15:15) and 600 g K_2_O (Potassium Oxide) per tree, twice in the year, June and September. Irrigation was done during the long dry season of November to February, ten liters of water was delivered at the base of the trees twice a week. The commercial farm, was located at Aba Oyo Village (Federal University of Technology, Akure, Nigeria), with topographical elevation between 260 and 470 m above sea level. The climatic condition of the area during the study was peak dry condition between early December and late February 2013. The unripe orange fruits (physiologically matured with green peel) with 9° Brix and ripe orange fruits (commercially matured with orange yellow peel) with 13° Brix, were picked early in the morning by 9 am. Sweet oranges with an average weight of 175.0 ± 2 g each were used; they were washed with distilled water, and then manually peeled. The obtained peels were air dried in a ventilated oven at 40 °C for 48 h, milled using a laboratory hammer mill (Mikro Samplmill Lab Hammer Mill, ¾ HP motor) and passed through a 24 mesh sieve size to produce the orange peel powder (OPP) [23]. OPP produced was packed in polythene and kept in a sealed container at 4 °C until a month later when it was analyzed.

### 2.3. Decoction

Dried OPP (5 g) was boiled in water (100 mL) for 10 min. After cooling, the sample was centrifuged (5000 rpm for 10 min) and the clear solution was recovered in a conical flask, and then rinsed to 100 mL with water. This sample was analyzed as such by HPLC-DAD.

### 2.4. HPLC-DAD Analysis

High performance liquid chromatography (HPLC-DAD) was performed with a Shimadzu Prominence Auto Sampler (SIL-20A) HPLC system (Shimadzu, Kyoto, Japan), equipped with Shimadzu LC-20AT reciprocating pumps connected to a DGU 20A5 degasser with a CBM 20A integrator, SPD-M20A diode array detector and LC solution 1.22 SP1 software.

#### Quantification of Compounds

Reverse phase chromatographic analyses were carried out under gradient conditions using C_18_ column (4.6 mm × 150 mm, 5 μm diameter particles, Dikma Technologies Inc., Beijing, China); the mobile phase was water containing 1% formic acid (A) and methanol (B), and the composition gradient was: 12% of B until 10 min and changed to obtain 20%, 30%, 50%, 60%, 70%, 20% and 10% B at 20, 30, 40, 50, 60, 70 and 80 min, respectively, following the method described by [24] with slight modifications. Orange peel (ripe and unripe) extracts and mobile phase were filtered through 0.45 μm membrane filter (Millipore) and then degassed by ultrasonic bath (25 °C; 10 min; 120Watts). Prior to use, the OPP extract was analyzed; at a concentration of 15 mg/mL. The flow rate was 0.6 mL/min, injection volume 50 μL and the wavelength were 280 nm for catechin and epicatechin, 327 nm for caffeic acid, and 365 nm for quercetin, quercitrin, kaempferol, luteolin, naringin and rutin. All the samples and mobile phase were filtered through 0.45 μm membrane filter (Millipore) and then degassed by ultrasonic bath prior to use. Stock solutions of standards references were prepared in the HPLC mobile phase at a concentration range of 0.025–0.250 mg/mL for quercetin, quercitrin, kaempferol, luteolin, naringin and rutin; and 0.030–0.350 mg/mL for caffeic acid, epicatechin and catechin. Chromatography peaks were confirmed by comparing its retention time with those of reference standards and by DAD spectra (200 to 500 nm). Calibration curve for catechin: *Y* = 13067 *X* + 1281.9 (*r* = 0.9997); epicatechin: *Y* = 11983 *X* + 1162.5 (*r* = 0.9999); caffeic acid: *Y* = 11961 *X* + 1187.0 (*r* = 0.9998); naringin: *Y* = 12647 *X* + 1346.9 (*r* = 0.9994); quercitrin: *Y* = 11893 *X* + 1187.6 (*r* = 0.9997); rutin: *Y* = 12706 *X* + 1384.5 (*r* = 0.9999), kaempferol: *Y* = 13509 *X* + 1305.8 (*r* = 0.9991), luteolin: *Y* = 11865 *X* + 1259.6 (*r* = 0.9998) and quercetin: *Y* = 13507 *X* + 1271.9 (*r* = 0.9997). All chromatography operations were carried out at ambient temperature and in triplicate.

The limit of detection (LOD) and limit of quantification (LOQ) were calculated based on the standard deviation of the responses and the slope using three independent analytical curves. LOD and LOQ were calculated as 3.3 and 10 σ/S, respectively, where σ is the standard deviation of the response and S is the slope of the calibration curve [25].

### 2.5. In vitro Antioxidant Studies of Unripe and Ripe Orange Peel

#### 2.5.1. Determination of Total Phenol Content

The total phenol content was determined as suggested by Pereira *et al.* [26]. Briefly, appropriate dilutions of the extracts were oxidized with 2.5 mL 10% Folin-Ciocalteau’s reagent (v/v) and neutralized by 2.0 mL of 7.5% sodium carbonate. The reaction mixture was incubated for 40 min at 45 °C and the absorbance was measured at 765 nm using a Visible Spectrophotometer (Model 721 Visible Spectrophotometer, Axiom Mediral LMD, UK). The total phenol content was subsequently calculated as gallic acid equivalent.

#### 2.5.2. Determination of Total Flavonoid Content

The total flavonoid content of both extracts was determined using Folin Ciocalteu reagent as described by Singleton *et al.* [27]. About 0.5 mL of appropriately diluted sample was mixed with 0.5 mL methanol, 50 μL 10% AlCl_3_, 50 μL 1M potassium acetate and 1.4 mL water, and incubated at room temperature for 30 min. The absorbance of the reaction mixture was subsequently measured at 415 nm using a Visible Spectrophotometer (Model 721 Visible Spectrophotometer); the total flavonoid content was then calculated. The non-flavonoid polyphenols were taken as the difference between the total phenol and total flavonoid content.

#### 2.5.3. Determination of Ferric Reducing Antioxidant Property

The ferric reducing property of the extracts was determined by assessing the ability of the extract to reduce FeCl_3_ solution as described by Oyaizu [28]. About 2.5 mL aliquot was mixed with 2.5 mL 200 mM sodium phosphate buffer (pH 6.6) and 2.5 mL 1% potassium ferricyanide. The mixture was incubated at 50 °C for 20 min. and then 2.5 mL 10% trichloroacetic acid was added. This mixture was centrifuged at 650 rpm for 10 min and 5 mL of the supernatant was mixed with an equal volume of water and 1 mL of 0.1% ferric chloride. The absorbance was measured at 700 nm using a Visible Spectrophotometer (Model 721 Visible Spectrophotometer), and the ferric reducing antioxidant property was subsequently calculated.

#### 2.5.4. ABTS* Scavenging Ability

The ABTS* scavenging ability of both extracts were determined according to the method described by Re *et al.* [29]. The ABTS* was generated by reacting an (7 mmol/L) ABTS aqueous solution with K_2_S_2_O_8_ (2.45 mmol/L, final concentration) in the dark for 16 h and adjusting the Abs at 734 nm to 0.700 with ethanol. A measurement of 0.2 mL of both solvent extracts at different concentrations was added to 2.0 mL ABTS* solution and reaction mixture was allowed to stand at 30 °C the absorbances were measured using a Visible Spectrophotometer (Model 721 Visible Spectrophotometer) at 734 nm after 15 min. The trolox equivalent antioxidant capacity was subsequently calculated.

#### 2.5.5. DPPH Free Radical Scavenging Ability

The free radical scavenging ability of the extracts against DPPH (1,1-diphenyl-2 picrylhydrazyl) free radical was evaluated as described by Gyamfi *et al.* [30]. Appropriate dilution of the extracts (1 mL) was mixed with 1 mL; 0.4 mM methanolic solution containing DPPH radicals, the mixture was left in the dark for 30 min and the absorbance was measured using a Visible Spectrophotometer (Model 721 Visible Spectrophotometer) at 516 nm. The DPPH free radical scavenging ability was subsequently calculated.

#### 2.5.6. Fenton Reaction (Degradation of Deoxyribose)

The ability of the extract to prevent Fe^2+^/H_2_O_2_ induced decomposition of deoxyribose was determined as described by Halliwell *et al.* [31]. The extract 0–100 μL was added to a reaction mixture containing 120 μL of 20 mM deoxyribose, 400 μL of 0.1 mM phosphate buffer, 40 μL of 500 mM of FeSO_4_, and the volumes were made up to 800 μL with distilled water. The reaction mixture was incubated at 37 °C for 30 min and the reaction was then stopped by the addition of 0.5 mL of 28% trichloro acetic acid. This was followed by addition of 0.4 mL of 0.6% thiobarbituric acid solution. The tubes were subsequently incubated in boiling water for 20 min. The absorbance was measured at 532nm using a Visible Spectrophotometer (Model 721 Visible Spectrophotometer).

#### 2.5.7. Fe^2+^ Chelation Assay

The Fe^2+^ chelating ability of both extracts were determined using a modified method of Minotti and Aust, [32]. Freshly prepared 500 μM FeSO_4_ (150 μL) was added to a reaction mixture containing 168 μL 0.1 M Tris-HCl (pH 7.4), 218 μL saline and the extracts (0–25 μL). The reaction mixture was incubated for 5 min, before the addition of 13 μL 0.25% 1,10-phenanthroline (w/v). The absorbance was subsequently measured at 510 nm using a Visible Spectrophotometer (Model 721 Visible Spectrophotometer). The Fe(II) chelating ability was subsequently calculated.

#### 2.5.8. Lipid Peroxidation Assay

The rats were decapitated under mild diethyl ether anaesthesia and the pancreas was rapidly isolated and placed on ice and weighed. This tissue was subsequently homogenized in cold saline (1/10, w/v) with about 10-up-and-down strokes at approximately 1200 r/min in a Teflon glass homogenizer (Glas-Col Co., CA, USA). The homogenate was centrifuged for 10 min at 3000× *g* to yield a pellet that was discarded, and a low-speed supernatant (S1) was kept for lipid peroxidation assay [33].

The lipid peroxidation assay was carried out using the modified method of Ohkawa *et al.* [34], 100 μL S1 fraction was mixed with a reaction mixture containing 30 μL of 0.1 M pH 7.4 Tris-HCl buffer, extract (0–100 μL) and 30 μL of 250 μM freshly prepared FeSO_4_. The volume was made up to 300 μL by water before incubation at 37 °C for 1 h. The color reaction was developed by adding 300 μL of 8.1% SDS (sodium doudecyl sulphate) to the reaction mixture containing S1; this was subsequently followed by the addition of 600 μL of acetic acid/hydrochloric acid at ratio 8:3 and then adjusted to pH 3.4. Added to the mixture was 600 μL of 0.8% TBA (thiobarbituric acid), this mixture was incubated at 100 °C for 1 h. TBARS (thiobarbituric acid reactive species) produced were measured at 532 nm and the absorbance was compared with that of standard curve using MDA (malondialdehyde).

### 2.6. Statistical Analysis

All samples for the phenolic profile were analyzed in triplicate and the results are expressed as mean ± standard deviations (SD) of three determinations. Averages followed by different letters in the column differ by Tukey test at *p* < 0.05. Values with different alphabet along the same row are significantly different (*p* < 0.05) by one-way analysis of variance (ANOVA).

Data for the *in vitro* antioxidant activities were analyzed and expressed as mean ± standard error mean (S.E.M.). The statistical analysis was carried out using one-way analysis of variance (ANOVA) and means were separated using Duncan multiple range test (DMRT), *p* < 0.05 were considered as significant. The EC_50_ was determined using linear regression analysis for the antioxidant assay.

## 3. Results and Discussion

### 3.1. Identification and Quantification of Phenolics Using HPLC-DAD

**Figure 1:**
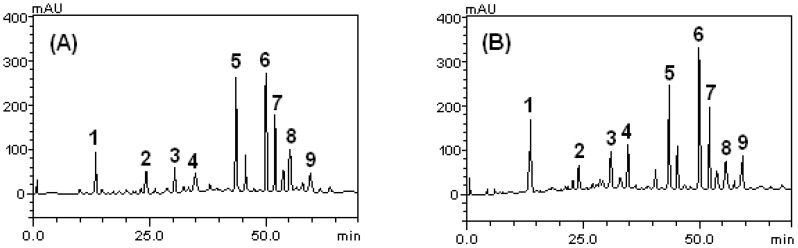
Representative high performance liquid chromatography profile of unripe orange peel powder (**A**) and ripe orange peel powder (**B**). Catechin (peak 1), caffeic acid (peak 2), naringin (peak 3), epicatechin (peak 4), rutin (peak 5), quercitrin (peak 6), quercetin (peak 7) kaempferol (peak 8) and luteolin (peak 9).

HPLC-DAD phenolic compounds analysis of orange peel (unripe and ripe) extracts revealed the presence of catechin (*t*_R_ = 14.37 min; peak 1), caffeic acid (*t*_R_ = 24.79 min; peak 2), naringin (*t*_R_ = 31.25 min; peak 3), epicatechin (*t*_R_ = 34.97; peak 4), rutin (*t*_R_ = 43.81 min; peak 5), quercitrin (*t*_R_ = 49.68 min; peak 6), quercetin (*t*_R_ = 52.41 min; peak 7), kaempferol (*t*_R_ = 56.11 min; peak 8) and luteolin (*t*_R_ = 58.39 min; peak 9) (Table 1 and Figure 1). A total of nine phenolic compounds which included four flavonoids, were identified and quantified as shown in Table 1. The chromatographic parameters of the method used for quantification of the compounds are shown in Table 2. Among the phenolic compounds, quercitrin and rutin showed highest concentration in both unripe and ripe peel with 18.79 mg/g and 18.6 mg/g obtained for unripe orange peel while 22.61 mg/g and 17.93 mg/g were obtained for ripe orange peel. Rutin, also called rutoside, quercetin-3-*O*-rutinoside, and sophorin, was the glycoside between the flavonol quercetin and the disaccharide rutinose α-l-rhamnopyranosyl-(1→6)-β-d-glucopyranose. Rutin, a bioflavanoid glycoside with strong antioxidant properties was found in many plants fruits and fruit rinds (especially the citrus fruits; orange, grapefruit, lemon, and lime), and apple and berries. Rutin (quercetin rutinoside), like quercitrin, is a glycoside of the flavonoid quercetin. As such, the chemical structures of both are very similar, with the difference existing in the hydroxyl functional group. Both quercetin and rutin are used in many countries as medications for blood vessel protection, and are ingredients of numerous multivitamin preparations and herbal remedies. Quercitrin has been reported to have antioxidant and anti-carcinogenic qualities via its inhibition of neoplastic transformation by blocking activation of the Mitogen-Activated Protein Kinase (MAPK) pathway and stimulation of cellular protection signaling [35]. It was observed from the present investigation that quercitrin and rutin concentration in ripe orange peel powder was higher than that of unripe orange peel powder. Reports on olive fruits showed that the phenolic contents have direct effect on the chemical composition of olive products which was dependent on the cultivar, the pedoclimatic production conditions, the agronomic techniques, and fruit ripening [36,37,38,39]. This result was in agreement with the report of [2], who observed that ripe raspberries (100% maturity) had stronger antioxidant activities and higher total anthocyanin content when compared with the pink stage (50% maturity). On the contrary, the report of [40] in tomato phenolics revealed lower quercitrin and rutin in ripe tomato. However, the observation suggested that OPP powder especially the ripe one might be used in the promotion of cardiovascular health.

**Table 1:** Phenolic acids and flavonoids composition of orange peel powder (ripe and unripe) extracts.

Compounds	Unripe Orange Peel (mg/g)	Ripe Orange Peel (mg/g)
Catechin	5.91 ± 0.03 ^c,B^	12.49 ± 0.01 ^c,A^
Caffeic acid	3.45 ± 0.01 ^d,A^	3.57 ± 0.02 ^e,A^
Naringin	3.58 ± 0.02 ^d,B^	5.71 ± 0.02 ^d,A^
Epicatechin	3.21 ± 0.01 ^d,B^	6.08 ± 0.01 ^d,A^
Rutin	18.65 ± 0.03 ^a,A^	17.93 ± 0.03 ^b,B^
Quercitrin	18.77 ± 0.01 ^a,B^	22.61 ± 0.01 ^a,A^
Quercetin	10.39 ± 0.01 ^b,B^	14.03 ± 0.02 ^b,A^
Kaempferol	5.86 ± 0.02 ^c,A^	3.76 ± 0.03 ^e,B^
Luteolin	3.19 ± 0.03 ^d,B^	5.83 ± 0.01 ^d,A^

Results are expressed as mean ± standard deviations (SD) of three determinations. Values with different alphabet along the same column (lowercase letters) are significantly different by Tukey test (*p* < 0.05). Values with different alphabet along the same row (capital letters) are significantly different (*p* < 0.05) by one-way analysis of variance (ANOVA).

**Table 2:** Chromatographic parameters of the method used for quantification of compounds in orange peel powder (ripe and unripe) extracts.

Compounds	Concentration Range mg/mL	Standard Curves	Correlation Coefficient (r)	LOD µg/mL	LOQ µg/mL
Catechin	0.030–0.350	*Y* = 13067 *X* + 1281.9	0.9997	0.017	0.056
Caffeic acid	0.030–0.350	*Y* = 11961 *X* + 1187.0	0.9998	0.026	0.090
Naringin	0.025–0.250	*Y* = 12647 *X* + 1346.9	0.9994	0.011	0.037
Epicatechin	0.030–0.350	*Y* = 11983 *X* + 1162.5	0.9999	0.032	0.105
Rutin	0.025–0.250	*Y* = 12706 *X* + 1384.5	0.9999	0.028	0.096
Quercitrin	0.025–0.250	*Y* = 11893 *X* + 1187.6	0.9997	0.009	0.034
Quercetin	0.025–0.250	*Y* = 13507 *X* + 1271.9	0.9997	0.019	0.063
Kaempferol	0.025–0.250	*Y* = 13509 *X* + 1305.8	0.9991	0.021	0.069
Luteolin	0.025–0.250	*Y* = 11865 *X* + 1259.6	0.9998	0.035	0.118

LOD, limit of detection; LOQ, limit of quantification.

### 3.2. In vitro Antioxidant Studies of Unripe and Ripe Orange Peel

Table 3 reveals the antioxidant properties (total phenols, total flavonoids, ABTS*, and FRAP) in OPP. The total phenolic and total flavonoid contents of ripe orange peel (9.40 mg GAE/g and 4.20 mg QE/g) were significantly higher than values obtained for the unripe orange peel of 5.27 mg GAE/g and 3.20 mg QE/g respectively. Phenolic compounds are important fruit constituents because they exhibit antioxidant activity by inactivating lipid free radicals or preventing decomposition of hydroperoxides into free radicals [41]. The phenolic contents obtained in the peels are higher than values obtained for orange juice [42] and some commonly consumed leafy vegetables in Nigeria [43,44]. Polyphenols such as flavanol contributed significantly to the ferric reducing capacity and radical scavenging capacity of orange peel during ripening, as ripe orange peel exhibited higher ABTS* (16.89 mmol/TEAC g) and FRAP (91.38 mg/GAE100 g) compared to the unripe orange peel. Peel extract gave a remarkable effect in protecting cells from oxidative damage [45], this could be due to the additional phenolic compounds present in peel compared to juice and pulp residue [46]. It implies therefore that the antioxidant capacities of orange peel increased significantly during ripening as the ripe peel exhibited higher antioxidant activities and contents than the unripe peel.

**Table 3:** Antioxidant properties of unripe and ripe orange peel powder.

ANTIOXIDANT PROPERTIES	UNRIPE	RIPE
Total phenol content (mgGAE/g)	5.27 ± 0.03 ^b^	9.40 ± 0.01 ^a^
Total flavonoid content (mgQE/g)	3.30 ± 0.30 ^b^	4.20 ± 0.02 ^a^
ABTS* scavenging ability (mmol/TEAC g)	14.68 ± 0.01 ^b^	16.89 ± 0.02 ^a^
FRAP (mg/GAE100 g)	70.69 ± 0.01 ^b^	91.38 ± 0.01 ^a^

Values represent means ± standard deviation of triplicate (*n* = 3). Values with different alphabet along the same row (for the same antioxidant properties) are significantly different (*p* > 0.05).

Table 4 shows the EC_50_ of some antioxidant properties. EC_50_ (implies concentration required to obtain a 50% antioxidant effect) it is a typically used parameter to express the antioxidant capacity and to compare the activity of different compounds. A lower value of EC_50_ corresponds to a higher antioxidant activity of the orange peel extract. Free radical scavenging expressed as EC_50_ were 2.71 (unripe) and 2.23 mg/mL DPPH for ripe, the most effective being ripe orange peel. The trend in the results agree with the phenolic distribution in the citrus peels (Table 1) and other earlier reports, where correlations were reported between phenolic content and antioxidant capacity of some plant foods [42,45,46]. DPPH free radical scavenging ability is frequently used in the determination of free radical scavenging ability; however it has the limitation of color interference and sample solubility [47]. Ripe orange peel showed the highest scavenging activity against hydroxyl radicals (OH*), Fe^2+^ chelation, and malondialdehyde with EC_50_ values of 0.57, 0.49 and 0.50 mg/mL, respectively when compared with the unripe orange peel. The Fe^2+^ chelating ability of the ripe orange peel (EC_50_ = 0.49 mg/mL) is higher than their corresponding OH* scavenging abilities (EC_50_ = 0.57 mg/mL) as presented in Table 4; these findings agree with some earlier reports on the OH* and Fe^2+^ chelating abilities of phenolics of some plant foods such as leafy vegetables, peppers, and spices that have stronger Fe^2+^ chelating than OH* scavenging abilities [48,49]. The Fe^2+^ chelating ability of the phenolics could be attributed to the presence of two or more of the following functional groups namely, OH, -SH, -COOH, PO_3_H_2_, C=O, -NR_2_, -*S*- and -*O*- in a favourable structure-function configuration [50,51,52]. MDA formation is used as the general indicator of the extent of lipid peroxidation resulting from oxidative stress. It increases when Fe^2+^ induces lipid peroxidation by catalyzing the decomposition of hydrogen peroxide to generate hydroxyl radical via the Fenton reaction [53]. The ripe peel significantly (*p* < 0.05) inhibited MDA production in the pancreas in a dose-dependent manner, ripe peel had the highest inhibitory effect on MDA production in the pancreas (*in vitro*), while the unripe had the least inhibitory effect (Table 4). The possible mechanism through which the ripe peel of orange protects the pancreas could be by Fe^2+^chelation [54] and the scavenging of OH* [49,54,55] abilities. Since the phenolic extracts had higher Fe^2+^ chelating ability than OH* scavenging ability, Fe^2+^ chelation could be the domineering mechanism through which the phenolics protect pancreas membrane from Fe^2+^ induced lipid peroxidation in the pancreas.

**Table 4:** EC_50_ (mg/mL) of ripe and unripe orange peel on different antioxidant parameters.

ANTIOXIDANT PARAMETERS	UNRIPE	RIPE
DPPH*	2.71 ± 0.03 ^a^	2.23 ± 0.03 ^b^
OH*	0.67 ± 0.02 ^a^	0.57 ± 0.03 ^b^
Fe^2+^ Chelation	0.57 ± 0.02 ^a^	0.49 ± 0.01 ^b^
Malondialdehyde (MDA)	0.63 ± 0.06 ^a^	0.50 ± 0.01 ^b^

Values represent means ± standard deviation of triplicate (*n* = 3). Values with the different alphabet along the same row (for the same parameter) are significantly different (*p* > 0.05).

## 4. Conclusions

According to HPLC-DAD analysis for the identification and quantification of phenolics, quercitrin and rutin were the most predominant phenolics. The *in vitro* antioxidant activities of the extracts of sweet orange peel revealed the radical scavenging activity of the unripe and ripe orange peel, which is strongly associated with the presence of flavonoids, phenolic acids, and their derivatives, thereby supporting the relevance of sweet orange peel as important dietary source of antioxidant compounds. Orange peel powder, especially the ripe orange peel may therefore be exploited as natural antioxidant in food application as well as for health supplements or functional food, to alleviate oxidative stress.
